# The assessment of risk factors for the Central/East African Genotype of chikungunya virus infections in the state of Kelantan: a case control study in Malaysia

**DOI:** 10.1186/1471-2334-13-211

**Published:** 2013-05-08

**Authors:** Ahmad Faudzi Yusoff, Amal Nasir Mustafa, Hani Mat Husaain, Wan Mansor Hamzah, Apandi Mohd Yusof, Rozilawati Harun, Faezah Noor Abdullah

**Affiliations:** 1 Institute for Medical Research, National Institutes of Health, Ministry of Health, Jalan Pahang, Kuala Lumpur 50588, Malaysia; 2 Kelantan State Health Department, Ministry of Health Malaysia, Aras 5, Wisma Persekutuan Kota Bharu, Jalan Bayam, Kota Bharu, Kelantan, 15590, Malaysia

**Keywords:** Chikungunya, Case–control study, Central/East African genotype, Risk factors

## Abstract

**Background:**

The aims of the study were to assess the risk factors in relation to cross border activities, exposure to mosquito bite and preventive measures taken.

An outbreak of chikungunya virus (CHIKV) infection in Malaysia has been reported in Klang, Selangor (1998) and Bagan Panchor, Perak (2006). In 2009, CHIKV infection re-emerged in some states in Malaysia. It raises the possibilities that re-emergence is part of the epidemics in neighbouring countries or the disease is endemic in Malaysia. For this reason, A community-based case control study was carried out in the state of Kelantan.

**Methods:**

Prospective case finding was performed from June to December 2009. Those who presented with signs and symptoms of CHIKV infection were investigated. We designed a case control study to assess the risk factors. Assessment consisted of answering questions, undergoing a medical examination, and being tested for the presence of IgM antibodies to CHIKV. Descriptive epidemiological studies were conducted by reviewing both the national surveillance and laboratory data. Multivariable logistic regression analysis was performed to determine risk factors contributing to the illness. Cases were determined by positive to RT-PCR or serological for antibodies by IgM. CHIKV specificity was confirmed by DNA sequencing.

**Results:**

There were 129 suspected cases and 176 controls. Among suspected cases, 54.4% were diagnosed to have CHIKV infection. Among the controls, 30.1% were found to be positive to serology for antibodies [IgM, 14.2% and IgG, 15.9%]. For analytic study and based on laboratory case definition, 95 were considered as cases and 123 as controls. Those who were positive to IgG were excluded. CHIKV infection affected all ages and mostly between 50–59 years old. Staying together in the same house with infected patients and working as rubber tappers were at a higher risk of infection. The usage of Mosquito coil insecticide had shown to be a significant protective factor. Most cases were treated as outpatient, only 7.5% needed hospitalization. The CHIKV infection was attributable to central/east African genotype CHIKV.

**Conclusions:**

In this study, cross border activity was not a significant risk factor although Thailand and Malaysia shared the same CHIKV genotype during the episode of infections.

## Background

Chikungunya infection is a mosquito-borne disease caused by Chikungunya virus (CHIKV), an alphavirus belongs to the Togaviridae family. It is transmitted to humans by the bite of infected mosquitoes, usually the genus Aedes [1,2]. CHIKV infection was first described in Tanzania, Africa following an outbreak in 1952 [1-5] on the Makonde Plateau, along the border between Tanganyika and Mozambique [1]. The virus was first isolated in Tanzania in 1953 [2,6,7]. Chikungunya fever is an acute illness characterised by acute onset of fever, incapacitating and debilitating arthalgias, and sometimes arthritis, accompanied by conjunctivitis and petechial or maculopapular rash, and lasting for a period of 1–7 days [1,4,5,7]. The incubation period is usually 2–3 days with the range of 1–12 days. Some descriptions on chikungunya in local dialect, Makonde root verb Kungunyala, giving the meaning of “to become contorted” [1] or from the local dialect, Swahili it means “that which bend up” [4,8] which describing contorted posture of patients suffering severe joint pain associated with disease or stooped posture adopted by the patient as a result of the arthritis symptoms that the patient develops. It is a self-limiting illness and full recovery except 3 to 5% have prolonged arthritis up to 6 months [1]. The joint pain usually involves multiple joints and predominantly affects the small joints of hand, wrists, ankles and feet. Larger joints are much less involved [4,6]. Sometimes the joint swelling is associated with pain on movement and is worse in the morning. Many patients present with maculopapular rash with a flush over the face and trunk. Pruritis may be present. Some patients have been described to have photophobia, conjuctival injection and retro-orbital pain. Treatment is usually supportive towards the fever and joint pain. Definitive diagnosis is made only by laboratory testing. CHIKV infection is probably often undiagnosed or misdiagnosed as dengue due to some similarities in clinical presentation but, unlike dengue, there is no hemorrhagic or shock syndrome forms. CHIKV infection is well known from epidemics in Africa. Since then, CHIKV has caused occasional epidemics in sub-Sahara Africa and Asia. Asia has been recently responsible for severe epidemic reported in Indian Ocean from 2004–2007, which has caused several serious health and economic problems [9]. In 2005, an outbreak was reported on French island of ReUnion in the Indian Ocean [8,10]. In, 2006, Andhra Pradesh and similar outbreaks occurred in Bangalore, Tamil Nadu, Salem and Chennai. Other countries like Cambodia, Vietnam, Myanmar, Sri Lanka, India, Indonesia, Thailand, Philippines and Malaysia have also reported CHIKV infections [11]. In Malaysia an outbreak had been reported in 1998 in Klang, Selangor [12] and re-emerged in Bagan Panchor, Perak, 2006 [12,13] and Johor, 2008 [3]. It raises the possibilities that CHIKV infection is endemic in Malaysia or re-emergence of the infection is part of the epidemics in other neighbouring countries. A previous serological survey for alpha viruses conducted in Peninsular Malaysia showed that CHIKV antibody was detected in individuals in northern states bordering Thailand such as Perlis, Kedah and Kelantan in persons older than 20 years old [7]. The objectives of this study were to assess the risk factors in relation to cross border activities, exposure to mosquito bite and preventive measure taken.

## Methods

### Study design

A community-based case control study was conducted in four districts in the state of Kelantan which are bordering southern Thailand. They were Pasir Mas, Tanah Merah, Jeli and Tumpat. The study was carried out prospectively from June to December 2009. All hospitals and clinics in the four districts were asked to notify the suspected cases to their respective District Health Department. Patients presented with fever, rash and or joint pain were identified as suspected CHIKV infection cases. Confirmed CHIKV infection was defined as a person presented with clinical manifestation of CHIKV infection with positive Reverse transcription polymerase chain reaction (RT-PCR) or immunoglobulin (Ig) M antibodies detected in the sera. Controls were randomly selected using simple random sampling technique from healthy individuals in the affected community within the same enumeration blocks as a patient identified without signs and symptoms of CHIKV infection and negative laboratory results.

We had planned to recruit approximately one unmatched control for every case patient identified, regardless of whether or not the case met the confirmatory definition of CHIKV infection. We chose to control for age and gender during analysis rather than matching for these variables during enrolment, because of considerable logistic difficulties that we would have encountered in identifying appropriate matched controls. We determined the controls by negative results to confirmatory laboratory tests and exclude those whose serology results were positive to immunoglobulin (Ig) G. The structured questionnaires were prepared towards assessing the cross border activities, risk behaviour and risk of getting the infection. All cases and controls were face to face interviewed using the questionnaires. Demographic characteristics, exposure to mosquito bite and preventive measures used to avoid mosquito bites were also asked. The suspected cases which did not receive treatment were referred to Hospital for further management.

### Laboratory tests

Serum samples from suspected cases and those selected as controls were taken and subjected to a combination of three types of laboratory tests; virus isolation, molecular detection of CHIKV Ribonucleic acid (RNA) RNA by RT-PCR and assay of anti-CHIKV specific IgM and IgG. All specimens were sent to Institute for Medical Research, Kuala Lumpur for diagnostic confirmation. Suspected cases with a history of illness for 5 days or less, nucleic acid detection using PCR was tested and if more than 6 days of onset, the serological assay of anti-CHIKV IgM and IgG was performed by indirect immunofluorescent test. Cases were only determined by positive to RT-PCR or serological for antibodies by IgM. The serological assay anti- CHIKV IgG was also performed among the controls to exclude the possibilities of previous infections. CHIKV strain was confirmed by Deoxyribonucleic acid (DNA) sequencing [14].

### Data management and analysis

The epidemiological data and laboratory results were entered using Microsoft excel spreadsheet program. All data analysis was carried out using SPSS version 18 and Stata 11. The distribution of chikungunya cases were described by age, sex and localities. The descriptive results also included demographic and clinical presentation of patients. Analytical study was performed to determine the risk factors contributing to the illness. Univariate analysis was carried out for all risk factor variables. A multivariable logistic regression model was created by entering simultaneously all the risk factor variables. Multi-co-linearity and interaction were checked. The assessment of “goodness of fit” was done before the final model was established. The risk factors were assessed by crude odds ratio. Adjusted odds ratios were presented as significant findings.

The ethical approval was obtained from the Medical Research and Ethics Committee, Ministry of Health Malaysia and the study was funded by Ministry of Health Malaysia. Written informed consent was obtained from all participants prior to the interview.

## Results

### Descriptive study

**Figure 1:**
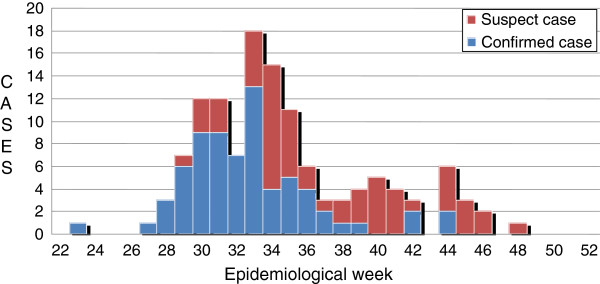
Number of Chikungunya infection among the suspected cases in, State of Kelantan, by epid week (22–52), 2009 (n=129).

From June to December 2009, a total of 129 suspected cases were reported in all four study districts. The number of suspected and confirmed cases by epidemiology weeks (22–52) is shown in Figure 1.

**Figure 2:**
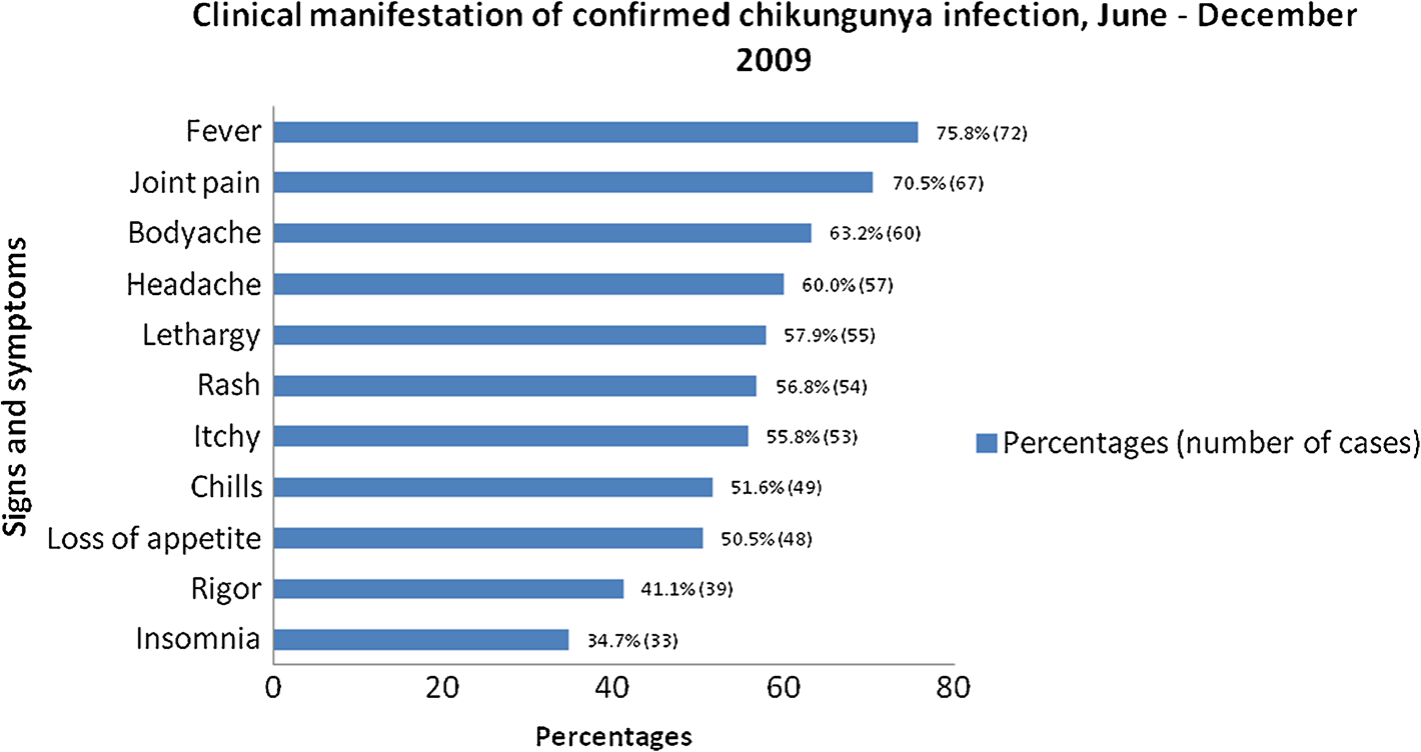
Clinical manifestation of confirmed chikungunya infection, June-December 2009.

Jeli district showed highest incidence, 64.2 per 100,000 compared to other districts; Tanah Merah (20.8 per 100,000), Pasir Mas (17.9 per 100,000) and Tumpat (0.6 per 100,000). Among the confirmed cases, they presented with fever (75.8%) followed by joint pain (70.5%), myalgia (63.2%), headache (60.0%) and rashes (56.8%) and others as shown in Figure 2. Knee joints were mostly affected (63.2%), followed by ankle (62.1%), wrist (57.9%), interphalengeal joint of hands (57.9%), elbow (49.5%), shoulder (49.1%) and others (14.7%).

Almost all cases with signs and symptoms of CHIKV infection sought treatment at clinics. About 75.7% (n=54) received treatment as out-patients. Only 21.4% (n=15) were admitted to hospital. Those who sought treatment as outpatients, 44.3% (n=32) went to government clinics and 31.4% (n=22) to private clinics. No death was attributed to CHIKV infection among the cases. CHIKV infection affected all ages and adults showed the highest percentage. The mean age was 41.5 years old, (SD: 15.97 range between 11 to 80 years old). Females (60%) shown to be higher in numbers than males (40%). Most of the cases were in the rural areas (96.8%). In 64.2% (n=61) of the cases, the homes were located in plantation areas in which 60.6% were in fruit farm (n=37), 13.1% in rubber estate (n=8) and 26.2% in mixed plantation areas (n=16). In 21.1% (n=20) of the cases, the houses were located within 200 meter from the plantation areas. The Malays (97.9%) were most affected followed by Chinese (1.1%) and others (1.1%). Housewives (32.6%) were affected the most followed by rubber tapper (22.1%), self employed (20.0%), student (7.4%) and others (6.3%) as shown in Table 1.

**Table 1:** Demographic characteristic of the cases and controls

**Demographic characteristic**		**Case**	**Control**	**Total**
Gender	Male	38(40.0%)	51(41.5%)	89(40.8%)
	Female	57(60.0%)	72(58.5%)	129(59.2%)
	Total	95(100%)	123(100%)	218(100%)
Age group	Adolescent	8(8.4%)	22(17.9%)	30(13.8%)
	Adult	73(76.8%)	84(68.3%)	157(72.0%)
	Elderly	14(14.2%)	17(13.8%)	31(14.2%)
	Total	95(100%)	123(100%)	218(100%)
Ethnic group	Malays	93(97.9%)	120(97.6%)	213(97.7%)
	Chinese	1(1.1%)	2(1.6%)	3(1.4%)
	others	1(0.8%)	1(1.1%)	2(0.9%)
	Total	95(100%)	123(100%)	218(100%)
Locality	Rural	92(96.8%)	114(92.7%)	206(94.5%)
	Urban	3(3.2%)	9(7.3%)	12(5.5%)
	Total	95(100%)	123(100%)	218(100%)
Occupation	Government officer	2(2.1%)	3(2.4%)	5(2.3%)
	Teacher	3(3.2%)	2(1.6%)	5(2.3%)
	Retired	1(1.1%)	1(0.8%)	2(0.9%)
	Student	7(7.4%)	18(14.6%)	25(11.5%)
	Self employed	19(20.0%)	23(18.7%)	42(19.3%)
	Labourer	5(5.3%)	8(6.5%)	13(6.0%)
	Others	6(6.3%)	15(12.2%)	21(9.6%)
	Housewife	31(32.6%)	46(37.4%)	77(35.3%)
	Rubber tapper	21(22.1%)	7(5.7%)	28(12.8%)
	Total	95(100%)	123(100%)	218(100%)
Level of education	Primary School	26(27.4%)	32(26.0%)	58(26.6%)
	Lower secondary school	27(28.4%)	34(27.6%)	61(28.0%)
	Upper secondary school	26(27.4%)	40(32.5%)	66(30.3%)
	College/Universities	1(1.1%)	6(4.9%)	7(3.2%)
	Religious School	4(4.2%)	4(3.3%)	8(3.7%)
	Not schooling	8(8.4%)	7(5.7%)	15(6.9%)
	No formal education	3(3.2%)	0(.0%)	3(1.4%)
	Total	95(100%)	123(100%)	218(100%)

### Laboratory results

We identified 305 persons from whom blood samples were collected. A total of 129 serum samples from the suspected cases and 176 from controls were tested. The results showed that among the suspected cases, 70 (54.3%) cases were diagnosed to have CHIKV infection based on laboratory confirmation [14]. Fifty nine (45.7%) suspected cases were negative. Out of 70 positive cases, 49 (70%) were positive by RT-PCR and 21 cases (30%) positive to IgM. Among the controls, 53 (30.1%) were found to be positive to serological for antibodies, 25 (14.2%) were IgM positive and 28 (15.9%) were positive to IgG. CHIKV infection was implicated in this study by the presence of IgG antibody alone and the controls which were positive to IgG were excluded from analytic analysis. Laboratory findings identified 14.2% ‘asymptomatic’ controls as confirmed CHIKV infections. Therefore, we identified the total number of confirmed cases which met the definition criteria as cases were 95 and 123 as controls as shown in Table 2. The DNA sequencing of the CHIKV was found to be the Central/East African genotype.

**Table 2:** Laboratory result of CHIKV infection in suspected cases and controls

**Laboratory test**	**Suspected case**	**Control**	**Total**
RT-PCR	**49(37.9%)**	-	49(16.1%)
IgM	**21(16.3%)**	**25(14.2%)**	46(15.1%)
IgG	-	28(15.9%)	28(9.2%)
Negative results	59(45.7%)	**123(69.9%)**	182(59.7%)
Total	129(100%)	176(100%)	305(100%)

RT-PCR: Reverse transcription polymerase chain reaction.

IgM: immunoglobulin (Ig)M.

IgG: immunoglobulin (Ig)G.

### Risk factors assessment

#### Univariate analysis

All demographic characteristics were not statistically significant as shown in Table 3. Staying together with relatives infected with CHIKV in the same house was a risk factor in getting the disease (OR=3.24, 95% CI: 1.82, 5.78 , p<0.0001) and had history been to potential places for mosquito bite (OR=3.06, 95% CI: 1.32, 7.09 , p=0.009) were statistically significant findings. Other factors such as visit infected chikungunya patient and frequently stay outside the house during daytime were not statistically significant.

**Table 3:** Crude and adjusted odds ratio with 95% CI of risk factors for CHIKV infection

**Factors**	**Crude OR**	**(95% CI)**	**p-value**	**Adjusted OR**	**(95% CI)**	**p-value**
Gender:						
Male	0.94	0.55-1.62		0.87	0.49-1.57	p>0.05
Female	Reference					
Ethnic groups:						
Malay	0.78	0.04-12.55	p>0.05	0.54	0.03-9.10	p>0.05
Chinese	0.50	0.13-19.56	p>0.05	0.37	0.01-16.83	p>0.05
Others	Reference					
Age groups						
Elderly	2.26	0.77-6.63	p>0.05	1.81	0.58-5.68	p>0.05
Adult	2.39	1.00-5.69	p>0.05	1.94	0.77-4.86	p>0.05
Adolescent	Reference					
Occupations:						
Government officer	Reference					
Teacher	2.25	0.18-28.25	p>0.05	2.78	0.20-38.91	p>0.05
Retired	1.50	0.06-40.63	p>0.05	1.07	0.38-29.75	p>0.05
Student	0.58	0.08-4.27	p>0.05	0.99	0.13-7.76	p>0.05
Self employed	1.24	0.19-8.20	p>0.05	1.62	0.23-11.3	p>0.05
Labourer	0.93	0.11-7.72	p>0.05	1.41	0.16-12.58	p>0.05
Others	0.60	0.08-4.54	p>0.05	0.88	0.11-7.19	p>0.05
Housewife	1.01	0.16-6.04	p>0.05	1.49	0.22-9.97	p>0.05
Rubber tapper	4.50	0.60-32.70	p>0.05	3.68	1.43-9.47	0.007
Locality:						
Urban	Reference					
Rural	2.42	0.64-9.20	p>0.05	1.54	0.37-6.35	p>0.05
Level of education:						
Primary School	Reference					
Lower secondary school	0.98	0.47-2.01	p>0.05	0.80	0.367-1.74	p>0.05
Upper secondary school	0.80	0.39-1.64	p>0.05	0.59	0.27-1.30	p>0.05
College/Universities	0.21	0.28-5.40	p>0.05	0.20	0.02-1.84	p>0.05
Religious School	1.23	0.28-5.40	p>0.05	1.50	0.29-7.59	p>0.05
No formal education	1.41	0.45-4.39	p>0.05	1.16	0.35-3.84	p>0.05
Staying together with relatives being infected with Chikungunya	3.24	1.82-5.78	p>0.05	2.50	1.37-4.58	0.003
Visit chikungunya patient	1.73	0.90-3.34	p>0.05	0.78	0.33-1.82	p>0.05
Staying more outside the house in early morning and late afternoon	1.66	0.76-3.63	p>0.05	0.80	0.28-2.31	p>0.05
Had history been to potential places for mosquito bite	3.06	1.32-7.09	0.009	1.55	0.52-4.67	p>0.05
Apply one of the preventive methods	0.17	0.06-0.51	0.002	0.39	0.11-1.37	p>0.05
Wearing the long sleeve	0.61	0.35-1.06	p>0.05	0.85	0.43-1.68	p>0.05
Apply the repellent lotion	1.17	0.32-4.26	p>0.05	1.45	0.35-6.09	p>0.05
Use of mosquito coils insecticide	0.38	0.22-068	0.001	0.44	0.24-0.79	0.007
Using the Mosquito net	0.65	0.38-1.11	p>0.05	0.61	0.31-1.19	p>0.05
Had history of travelling during the last 2 week	0.99	0.42-2.38	p>0.05	1.30	0.48-3.53	p>0.05
Working in area of plantation border to southern Thailand	3.98	0.41-38.87	p>0.05	1.36	0.12-14.8	p>0.05
Had been visited by relative from Southern Thailand	2.62	0.23-29.37	p>0.05	1.57	0.14-17.9	p>0.05
Had been visited by anybody from any places	5.56	0.97-26.84	p>0.05	3.86	0.75-19.64	p>0.05

Note: No body made any visit to relative in southern Thailand and no any relatives in southern Thailand that they know had been infected with chikungunya infection.

Cross border activities was not a significant risk factor. Such activities were working in area of plantation border to southern Thailand (OR=3.98, 95% CI: 0.41, 38.87, p>0.05), had been visited by relative from Southern Thailand (OR=2.62, 95% CI: 0.23, 29.37, p>0.05) , had been visited by anybody from any places (OR=5.56, 95% CI: 0.97, 26.84, p>0.05) and visit any places two weeks prior to interview (OR=0.99, 95% CI: 0.42, 2.38, p>0.05). We also asked any visit made to relative in southern Thailand and any relatives in southern Thailand that they know had been infected with chikungunya infection. None of them had the above activities. On preventive measures taken in preventing mosquito bite, applied one of the preventive methods (OR=0.17, 95% CI: 0.06, 0.51, p=0.002) and using mosquito coil insecticide (OR=0.38, 95% CI: 0.22, 0.68, p<0.01) were statistically significant as a protective factor. Other preventive measures such as wearing long sleeve shirt, application of mosquito repellent and use of mosquito net at night during sleep were not statistically significant.

#### Multivariable analysis

After adjusted the OR, rubber tappers and staying together with infected relatives with CHIKV infection in the same house were still significantly associated with chikungunya infection. The use of mosquito coil insecticide was found to be a significant protective factor as shown in Table 3.

## Discussion

CHIKV infection is not a notifiable disease but notification of CHIKV infection is an administrative mandatory [4]. The diagnosis of CHIKV infection must be reported within 24 hours to the national level [15]. This diagnosis is based only on the clinical manifestation without laboratory confirmation. This surveillance method is useful at the national level and important to curb CHIKV infection outbreak and reduce the morbidity nationally. In early 2009, surveillance data showed an increasing number of CHIKV infections within the state of Kelantan. However, it dropped significantly to a low level towards the end of 2009. Being monitored by epidemiological week at the national level, an early control and preventive measures can be taken at the state level. There was a possibility that CHIKV infection in the state of Kelantan was controlled successfully.

The types of surveillance system at the state and district levels are supposed to capture more detail information of the suspected cases including the laboratory results. We found cases with incomplete information at the state and district levels and there were cases with missing laboratory results. It will be very useful to the state and district levels to keep complete records as they are important in the outbreak management and risk factor assessment. We noticed that the study will be more informative if it is conducted during the peak of the infection. Due to some logistic limitations, the study can only be started in June 2009 and we noticed that the number of cases had dropped significantly after that. This might be due to several preventive measures taken by the affected districts.

The active case finding that we had started in June indirectly had increased the number of cases as shown in the epidemic curve. The number of cases further reduced as prevention and control measures were actively carried out over that period of time. From June to December 2009, CHIKV infection was confirmed in 95 patients. Of these, we found not only cases presented as acutely symptomatic but some patients did not complain any signs or symptoms of chikungunya infections. The active and mobile age groups were the most affected and these findings are similar with the studies conducted in southern Thailand [16,17]. Most of the houses of the cases were located in the fruit farm and some were located in mixed plantation either fruit farm and rubber plantation. Collecting fruits in the back yard of their houses is common during that period of time. Since the fruit season started from month of June 2009, this activity might be one of the potential risks in getting bites by the infected mosquitoes. We found among the cases, housewife showed a higher percentage and working as a rubber tapper had a greater risk of getting the infection. All symptomatic confirmed cases had history of fever lasting 2 to 7 days. In some patients it was associated with chills and rigors. Multiple joints pain, myalgia or body ache were the common symptoms. We observed that knee was the most commonly affected joint. Other affected joints were interphalangeal joints of hands, wrists, ankles and feet as commonly seen in other studies [1-4,6,15,18]. The disease is normally self-limited and rarely fatal [1,19]. Our study shows that the infection had high burden of morbidity. Only 21% needed hospitalization and most of the patients seek treatment as out-patients for symptomatic treatment. No death was reported due to CHIKV infection during the study period. The CHIKV infection was significantly associated with staying together with infected relatives as we found in this study and similar as in a previous study [16]. Cross border activities were not significant risk factors in this study. Preventive precaution towards crossing border activities might be one of the possible reasons that the communities had taken since both countries had experienced the infection during the early year of 2009. The preventive measures taken by the health department in managing the vector borne diseases outbreak pertaining to chikungunya and dengue fever in early months of 2009 is a possible impact in stopping the transmission of the infection. Mosquito coil insecticide used shown to be significantly as a protective factor. Mosquito coil insecticide was commonly used by community either inside or outside the house. The reasons why mosquito coil insecticide is commonly used because it produces smoke which keep away the adult mosquitoes. Furthermore, it was cheaper compared to the other types of insecticides available such as repellent and spray. The CHIKV infection in non-immune population in the state of Kelantan was attributable to emerging Central/East African genotype. The similar genotype was found in two studies done in Narathiwat and Songkla, Thailand [16,17]*.* Both studies were conducted at the same time when the state of Kelantan experienced an increased number of chikungunya cases in early 2009.

### Limitations

Logistic problems in early phase of the study, support tools in sending specimens to laboratories in Kuala Lumpur, refusals to participate in the study by potential subjects, duration of study period and refusals for blood to be taken for specimen especially among younger subjects were among the limitations we faced during the study.

## Conclusion

Central/East African CHIKV genotype is a causative virus. The study showed that reducing exposure to the sick patient during the viraemic phases was among the ways to avoid further transmission especially during the outbreak period. Rubber tapper was an occupational risk factor of getting infection. Cross border activity was not a significant risk factor although Southern Thailand and Northern Malaysia districts shared the same CHIKV genotype during the episode of infection. Continuous preventive measures taken were important to reduce the transmission of infection and using the mosquito coil insecticide was found to be one of the significant preventive methods in this study.

## Abbreviations

CHIKV: Chikungunya virus; RT-PCR: Reverse transcription polymerase chain reaction (RT-PCR); IgM: immunoglobulin (Ig) M; IgG: immunoglobulin (Ig) G; DNA: Deoxyribonucleic acid.

## Competing interests

The authors declared that they have no competing interests.

## Authors’ contributions

The authors made substantial contributions to conception and design, acquisition of data, analysis and interpretation of data and were involved in drafting the manuscript and revising it critically for important intellectual content and gave final approval of the version to be published. Other authors participated sufficiently in the work to take public responsibility for appropriate portions of the content. MAY carried out the molecular genetic studies, participated in the sequence alignment and drafted the manuscript and also carried out the immunoassays. MAY participated in the sequence alignment. AFY, ANM, HH, WMH, RH, NFA participated in the design of the study and performed the statistical analysis. AFY, ANM, WMH, RH, NFA conceived of the study, and participated in its design and coordination and helped to draft the manuscript. All authors read and approved the final manuscript.

## Pre-publication history

The pre-publication history for this paper can be accessed here:

http://www.biomedcentral.com/1471-2334/13/211/prepub
